# Compression indexes measured by transthoracic echocardiographic might be not accurate without interrupting chest compressions

**DOI:** 10.1186/s13054-020-2748-3

**Published:** 2020-02-13

**Authors:** Li-Min Wang, You Zhong, Su Ming-Hua, Wu Meng-Jun

**Affiliations:** 1 Department of Gynecology, Chengdu Women’s and Children’s Central Hospital, University of Electronic Science and Technology, Chengdu, Sichuan China; 2 Department of Pediatric Surgery, Chengdu Women and Children’s Central Hospital, University of Electronic Science and Technology, Chengdu, Sichuan China; 3 Department of Emergency, Sichuan Academy of Medical Sciences & Sichuan Provincial People’s Hospital, Chengdu, Sichuan China; 4 Department of Anesthesiology, The Affiliated Hospital, School of Medicine, UESTC Chengdu Women’s and Children’s Central Hospital, Chengdu, 610092 Sichuan China

*To the Editor:*

We read with great interest the recent article by Skulec et al. [1] Their inspirational study on this important topic deserves further discussion. In particular, we have some concerns in regard to their study conclusion; the authors have claimed maximal compression index (CImax) is a candidate parameter for the guidance of hemodynamic-directed cardiopulmonary resuscitation (CPR).

Transthoracic echocardiography (TTE) has been proposed as a tool with the potential to improve outcomes in cardiac arrest [2]. An important limitation to the practice of TTE during cardiac arrest resuscitation is the technical difficulty in obtaining adequate cardiac windows during the seconds available during CPR pauses for rhythm checks. Huis et al. [2] demonstrated that TTE during cardiac arrest results in prolonged rhythm checks greater than the recommended American Heart Association (AHA) guidelines of 10 s. Other common impediments to cardiac assessment by TTE during arrest include external and patient-related factors such as defibrillator pads, automated compression devices, obesity, lung disease, and gastric insufflation [3].

In the study by Skulec et al., after the enrollment, transthoracic echocardiographic investigation was performed during ongoing chest compressions in all of the patients. However, an important question is raised, whether transthoracic echocardiographic investigation was accurate without interrupting chest compressions.

In addition, the right heart was considerably more difficult to visualize than the left heart during chest compression by TTE [4]. The tricuspid valve seemed the most inconstant in its motion during external chest compression and release. Most importantly, Werner et al. [4] could not see a significant diminution in overall left ventricular chamber size in the two patients in whom endocardial definition was adequate for examination in at least two tomographic planes during chest compression by TTE.

So, under this setting, we think that measurement of the maximal and minimal diameters of the right and left ventricles for calculation of compression indexes might be not accurate. From the discussion above, the current study by Skulec did not provide convincing evidence that the CImax has a reliable value as a candidate parameter for the guidance of hemodynamic-directed CPR.

## Authors’ response

Roman Skulec, Petr Vojtisek and Vladimir Cerny

Sir,

We do appreciate the comments of Wang et al. to our recent article regarding measurement of the hemodynamic efficiency of cardiopulmonary resuscitation (CPR) for out-of-hospital cardiac arrest by evaluation of the degree of compression of the right and left ventricles by transthoracic echocardiography (TTE) [1, 5].

They present that intra-arrest TTE evaluation makes relevant acquisitions difficult to obtain. Their opinion is supported by the small case series of five patients by Werner et al. [4].

We agree this is a key issue. Obtaining high-quality echocardiographic images of the heart during CPR is difficult, especially during ongoing chest compressions. This is confirmed by our findings. It was possible to acquire echocardiographic recordings of sufficient quality in only 18 of the 30 patients enrolled. On the other hand, there are some ways to increase the quality of recordings, and we applied them in every single patient. First, before starting echocardiographic recording for measurement of compression indexes, we were carefully looking for the optimal subcostal window during ongoing chest compressions, as long as necessary, to obtain high-quality recordings. This is significantly different from a conventional intra-arrest echocardiographic examination, where the time to perform is limited by a 10-s pause rhythm check. Second, we performed each measurement at the point where the aortic bulb disappears while tilting up the probe in the subcostal view.

We must also emphasize that implementation of new ultrasound imaging technologies made high-quality imaging by point-of-care ultrasound possible in more patients than in the past.

It would be nice to identify the key determinants behind poor-quality recordings in patients who were not included in the evaluation, but unfortunately, we do not have enough data to do that.

To summarize, despite all the relevant concerns of Wang et al., we consider the maximal compression index as a reliable candidate parameter for guiding CPR. However, this is applicable only in those patients where high-quality echocardiographic recordings can be obtained.

## Abbreviations

CPR: Cardiopulmonary resuscitation
TTE: Transthoracic echocardiography
